# Use of a Community Trail Among New and Habitual Exercisers: A Preliminary Assessment

**Published:** 2004-09-15

**Authors:** Paul M. Gordon, Samuel J. Zizzi, Jeff Pauline

**Affiliations:** West Virginia University, School of Medicine, Department of Human Performance and Exercise Science; West Virginia University, School of Physical Education, Morgantown, WVah; Ball State University, School of Physical Education, Muncie, Ind

## Abstract

**Introduction:**

We evaluated physical activity patterns and trail use among new and habitually active exercisers using onsite trail interviews.

**Methods:**

Using a cross-sectional study design, 414 adults who accessed two new trails that bisect a rural community of 26,809 residents were interviewed during the first summer of the trails' official operation (2001). The trails comprise 12 miles of level and paved surface and run parallel to adjacent water sheds, businesses, and neighborhoods. Recent trail activity patterns were obtained, including the following: frequency of use, mode of activity, duration, distance traveled on trail, access points, time of day used, use of exercise companions, and distance traveled to get to trail. Perceived enablers and barriers related to trail use were also obtained. Data were compared between newly adopted exercisers (new exercisers) and individuals active prior to development of the trails (habitually active exercisers).

**Results:**

Twenty-three percent of the trail users were new exercisers. New exercisers were more dependent on the trails as a primary outlet for physical activity than were habitually active exercisers (*P* < .001). New exercisers traveled shorter distances to access the trails and rated convenience as a primary reason for using them. Both safety and terrain issues emerged as enablers for trail use, and unsafe conditions emerged as a concern among new exercisers.

**Conclusion:**

A community trail may be an important vehicle for promoting physically active lifestyles. However, new exercisers must overcome issues of proximal and safe access from residential areas in addition to other safety concerns to achieve regular physical activity.

## Introduction

Although the health benefits of physical activity are now well established (1), 55% of Americans do not meet the minimal physical activity recommendations for health (2). Environmental and policy approaches to promoting physical activity have been recommended to change the physical and social environments that individuals inhabit. Public health officials theorize that when suitable facilities are available to community residents, physical activity levels increase (3,4). *Healthy People 2010* objectives recommend creating and enhancing access to places and facilities where people can be physically active (5). Furthermore, the Task Force on Community Preventive Services has recently issued a strong recommendation for policy and environmental approaches that create or enhance access to places for physical activity, along with information outreach activities, as an intervention to increase community physical activity levels (6).

One example of an environmental and policy approach to increase physical activity in the community is the development of a walking/bicycling trail. A community walking/bicycling trail can be a relatively low-cost intervention that may facilitate physical activity by reducing barriers related to cost, convenience, and accessibility (7,8). Moreover, because the trail is a permanent fixture within the community, it may facilitate the maintenance of a physically active lifestyle. Brownson et al examined the characteristics and possible impact of walking-trail development and suggested that walking trails may be particularly effective at reaching populations at high risk for inactive behaviors (9). Although recent studies have included trails as examples of physical environmental attributes of an active community (10), community walking/biking trails in particular have not been well studied. One recent investigation in Australia found that a newly constructed rail trail accompanied by a local promotional campaign increased cycling (11). More studies are needed to assess the importance of a community walking/biking trail on influencing physical activity levels.

It is not known how important a trail is among individuals who have newly adopted exercise habits. Nor is it known if the types of physical activity and patterns of trail use differ between new exercisers and habitually active exercisers. Although health officials have theorized that community recreation trails can provide convenient and accessible opportunities for engaging in regular physical activity, little data are available to describe the trails' importance, particularly among those who are transitioning toward an active lifestyle. In addition, the barriers and enablers to trail use, which may differ between new and habitually active exercisers, are important to understanding how to facilitate this transition. This information will provide health officials with insights that may be useful for promoting trail use and active lifestyles among residents within their communities.

## Methods

### Design

A cross-sectional study used data from an onsite interview survey of physical activity patterns, barriers, and enablers to trail use among adults using two new rail trails within the city of Morgantown, WVa.

The Caperton and Decker's Creek trails comprise 12 miles of paved trails that bisect the town and run adjacent to the Monongahela River and Decker's Creek, respectively. These trails also extend outside the city limits with an additional 14 miles of unpaved trails. Construction on these trails was completed in spring 2001. Rail trails are multiuse pathways constructed on abandoned railway beds and can be used for both recreational and transportation-related physical activity (12). In addition to stretching along waterways, these level trails intersect neighborhoods and business establishments within city limits.

### Sample

An interceptor-based survey approach was used instead of a population-based survey approach because of its better ability to identify and probe for trail users' perceptions and attitudes. Trained interviewers administered the Recreation Trail Evaluation Survey (RTES) to a sample of 414 adult trail users who lived in Monongalia County, West Virginia. Graduate students were trained to interview participants using skills training developed from other physical-activity interview-driven questionnaires (13). During training, interviewers reviewed and discussed the RTES questionnaire, rehearsed several practice interviews, and received grades on proficiency. Important features of the training sessions included clear explanations of the frame of reference for each question, how to control the pace and structure of the interview, and how and when to use prompts and other questions. To assure consistency, the same interviewers participated in the RTES pilot study prior to the study's initiation. Interviews were conducted two times per day using a randomized schedule that included predetermined blocks of time (7-10:00 AM, 11-2:00 PM, 3-6:00 PM, and 6-9:00 PM) and five different trail access points to ensure that samples fairly represented time of day, location on trail, and time of week (i.e., weekend vs weekday). The influence of weather was recognized as a possible limitation to data collection, but poor weather rarely occurred during data collection. The trail interview took approximately five to 10 minutes per participant to complete. Trail interviews took place for four weeks from June–July 2001. A true survey response rate (number of participants divided by total number of individuals who used the trails during the interview sessions) was not attained because of the way data were collected: some individuals may have passed by while interviews were being conducted. Nevertheless, 98% of all individuals who were approached were willing to participate. Willingness to participate in the study was high perhaps due to the novelty of newly developed trails in a smaller community and because the investigation took place shortly after their opening. Moreover, we did not infringe upon the participants' right to exercise. Rather, participants were interviewed as they entered or exited the various trailheads, and interviewers sometimes walked along with the exercisers during interviews. To prevent duplication, participants were asked at the start of the survey if they had previously been interviewed.

### Measures

The RTES measured recent trail physical activity patterns and included information on up to two types of physical activity performed on the trails (Appendix). The survey's exercise components queried participants on frequency and duration of activity, distance traveled on trail, and points of access for each type of activity. The question format used for the exercise components was similar to the format used by the Behavioral Risk Factor Surveillance System (BRFSS) (14). In addition, information was obtained on time of day, exercise companions, and method and distance traveled to get to the trail. Additionally, all respondents were asked if they participated in any of 10 non-trail physical activities in the previous month. These activities included walking, aerobic dance, bicycling, golfing, strength training, gardening, jogging/running, swimming/water exercises, organized team sports, and housework. Non-trail recreational patterns were assessed based on each activity's type, duration, and frequency.

In addition to self-reported distance traveled on the trail, actual distances were also calculated by premeasuring distances between access points and landmarks using an odometer wheel. Subjects were asked to identify points traveled on the trail (entry, turnaround, and exit) and the actual distance was calculated. Because there were no significant differences between self-reported and actual distance traveled on the trail, actual distance traveled is reported in the present study.

Each perceived enabler and barrier to trail use was measured using a five-point Likert scale ranging from 1 = not at all important to 5 = most important.Â  Enablers were defined as reasons for using the trail and included safety, scenery/environment, terrain (flat, paved), convenience, and atmosphere. Barriers were defined as items that may prevent participants from using the trail more and included safety issues, parking, accessibility, facilities, maintenance, and congestion. Using an open-ended question format, interviewers also asked participants to identify their primary enabler or barrier. Social and demographic information was collected on age, sex, marital status, race, employment status, educational attainment, and individual income level.

An initial pilot survey was developed from several existing documents that were obtained from similar studies (9,15) and tested over a three-week period. A sample was obtained at five key access points along the trail within the city limits to yield 161 users that included 90 female and 71 male adult respondents ranging in age from 18 to 82. Three expert reviewers analyzed results from the pilot survey to identify possible issues of clarity. Minor revisions to the trail user survey were made to address problems. While reliability measures are known for questions obtained from the BRFSS, no specific psychometric measures were obtained for the completed RTES survey. The finalized survey consisted of 33 closed and open-ended items.

Of primary interest to this investigation was to determine if the addition of the trail into the community caused any trail users to adopt new physical activity programs. Consequently, participants were asked, "Did you exercise regularly [more than three times per week for 20 minutes] before using this trail?" Three times per week was used as the frequency threshold for regular exercisers because of the associated health benefits that may exist among vigorous exercisers (1). This construct was designed to determine whether participants were currently engaged in a pattern of regular physical activity rather than to identify the prevalence of individuals meeting physical activity recommendations for health. Ninety-three (22.5%) trail users responded "no" to this question and were classified as new exercisers. The remaining 321 (77.5%) participants who answered "yes" were classified as habitually active exercisers. To determine differences that might exist between new exercisers and habitually active exercisers, comparisons of physical activity patterns and preferences for trail use were analyzed. Among all survey respondents, 94% were attaining 150 minutes of leisure-time physical activity per week, the amount recommended by the surgeon general (1).

This investigation was approved by the Human Subject's Institutional Review Board at West Virginia University.

### Analysis

Survey data were analyzed to determine the uses and usefulness of newly developed trails for physical activity within a community. The primary research question related to how many of the trail users in the sample were new exercisers and how many were habitually active prior to trail completion. After grouping participants, a series of analyses were conducted to explore potential demographic, behavioral, and motivational differences related to trail use between groups. All data were coded and entered into an SPSS 10.0 (SPSS Inc, Chicago, Ill) statistical software database for analyses. Chi-square analyses were conducted to determine differences in proportions. In addition, an independent *t*-test was used to test for differences in physical activity variables (e.g., frequency, duration, distance) between groups.

## Results

The sample (n = 414) was 94.4% white (n = 391), 44.9% male (n = 186), and 55.1% female (n = 228). Table 1 summarizes the primary demographic characteristics of the community trail users in this survey. These characteristics are representative of the community population. According to the 2000 U.S. Census, Monongalia County, West Virginia, is 93% white and 50% female (16). The age distribution for the county is as follows: 18–25 years = 22.0%; 26–35 years = 19.2%; 36–45 years = 17.8%; 46–64 years = 26.8%; and older than 65 years = 14.2%. The age distribution of the survey sample is comparable to the census distribution, except the sample had fewer respondents older than 65 (6.5%).

### Impact of trail on physical activity rates

Ninety-three (22.5%) trail users were classified as new exercisers, and 321 (77.5%) participants were classified as habitually active exercisers. A two-way chi-square analysis was performed to determine differences between groups across sex, age, and employment status. These analyses revealed no significant differences, suggesting that new exercisers and habitually active exercisers share similar demographic profiles. Analyses were also used to compare the frequency of additional physical activity reported between new and previously active exercisers. All respondents were asked if they had participated in any of 10 various physical activities (e.g., aerobic dance, swimming, team sports, housework, gardening) in the previous month. The total number of activities for each participant was computed, and an independent *t*-test was conducted to test the hypothesis. Habitually active exercisers reported significantly more frequency of additional physical activity (mean = 1.83 occurences; SD = 1.2) than new exercisers (mean = 1.2 occurences; SD = 1.1), *t* (412) = 4.51, *P* < .001. Additionally, more than twice as many new exercisers (31%) than habitually active exercisers (15%) reported that the trail was their only form of physical activity.

Nearly all (98%) of the new exercisers reported that their exercise amounts had increased when asked, "Since using the trail, has the amount of exercise that you do increased, decreased, or stayed the same?" Only 52% of the habitually active exercisers reported an increase. Conversely, 48% of habitually active exercisers and only 2% of new exercisers reported that their exercise amounts stayed the same. These data suggest that the physical activity patterns of nearly one half of habitually active exercisers were not impacted by the addition of the trail. Moreover, the perceived improvement in physical activity levels between new and habitually active exercisers was significantly different (*χ* ^2^[4] = 120.54, *P* < .001), with new exercisers reporting much greater increases in physical activity than habitually active exercisers with the addition of the trail (Figure).

**Figure:**
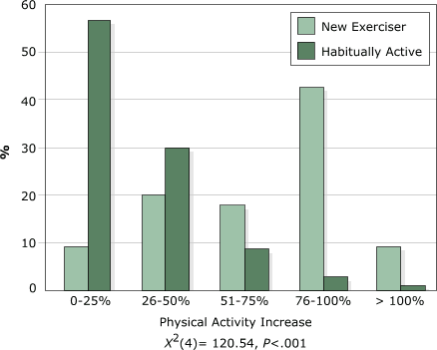
Percentage of increase in physical activity reported by new and habitually active exercisers when asked, "Since using the trail, approximately how much has your exercise level increased?"

**Table:** Χ2 (4) = 120.54, P < 0.001

	0-25%	26-50%	51-75%	76-100%	> 100%
New Exerciser	9.5	20.2	17.9	42.9	9.5
Habitually Active	56.8	30.1	8.7	3.1	1.3

### Types and patterns of physical activity on the trail

New exercisers traveled shorter distances to access the trails compared with habitually active exercisers (2.9 ± 3.4 miles vs 3.9 ± 6.0 miles; *P* = .03). The majority of respondents traveled to the trails by vehicle (81%). However, new exercisers were more likely to walk (18%) to the trails than habitually active exercisers (10.1%) (*P* = .04). Overall, these two groups differed in their patterns of physical activity on the trails. New exercisers were also more likely to walk (58% to 42%), less likely to run or jog (11% to 17%), and less likely to in-line skate (4% to 11%) than habitually active exercisers (*χ* ^2^[3] = 9.15, *P* = .02). Comparisons of average time and distance on the trails provide further support to the hypothesis that habitually active exercisers are engaging in different modes or higher exercise intensities compared with new exercisers. Habitually active exercisers traveled greater distances (*P* = .03) on the trail (6.64 ± 5.7 miles) than new exercisers (5.41 ± 3.7 miles) but did not spend a longer amount of time exercising (57.2 ± 30.1 min) than new exercisers (59.6 ± 30.2 min). Additionally, the frequency of weekly trail use averaged 3.4 (± 2.1) days per week in the entire sample. No significant difference in weekly trail use was observed between new exercisers (3.63 ± 1.5 days) and habitually active individuals (3.3 ± 2.3 days).

### Enablers and barriers to trail use

Table 2 presents the mean Likert-scale ratings of perceived enablers and barriers to trail use among new and habitually active exercisers. Participants were asked to rank each enabler and barrier, and rankings for enablers and barriers based on their aggregate level of importance were assigned by the investigators. New exercisers ranked enablers in the following order of importance: 1) convenience, 2) terrain, 3) safety, 4) scenery, and 5) atmosphere. In contrast, habitual exercisers ranked enablers in this order: 1) terrain, 2) convenience, 3) scenery, 4) safety, and 5) atmosphere. Mean ratings of enablers differed between groups. New exercisers rated safety (*P* = .03), terrain (*P* = .04), and convenience (*P* = .001) as significantly more important than habitually active exercisers. New exercisers rated unsafe conditions as a significantly higher barrier than habitually active exercisers (*P* = .04), although mean scores (3.1 ± 1.6) were in the middle of the five-point scale. All other perceived enablers and barriers were similar for both groups.

## Discussion

In this preliminary investigation, improvements in physical activity behavior occurred as a result of adding a community walking/biking trail, particularly among previously inactive participants. Approximately 25% of the trail users became regular exercisers (three or more times a week) as a result of the development of the trail. Moreover, new exercisers were much more dependent on the trail as a principal place for engaging in physical activity than those who exercised regularly prior to trail development. Thirty-one percent of new exercisers used the trail as the only venue for physical activity. This suggests that recreational trails may be a powerful vehicle for physical activity promotion, particularly among previously inactive individuals. Brownson et al suggested that within rural communities, sedentary individuals may be the most likely to benefit from walking trails (9). Although Morgantown, WVa, is a city of 26,809 residents, it is located in a rural region where there is little opportunity to safely engage in walking for physical activity. With narrow streets that lack traffic-calming strategies, bike lanes, and sidewalks, the community is not conducive for walking or bicycling. The introduction of a safe and convenient area to walk may be an excellent physical activity promotion tool. In a recent review of the effectiveness of interventions to increase physical activity, the *Guide to Community Preventive Services* proposed that creating access to places for physical activity, combined with informational outreach, is an effective means for increasing physical activity levels (6). The current investigation supports this recommendation.

New exercisers also traveled shorter distances to access the trail, implying that residential proximity to the trail may play an important role in whether individuals will use the trail. In further support of this, new exercisers were more likely to rate convenience as a primary reason for using the trail. Residential proximity to trails and their usage has previously been documented (10,11,17). Increases in self-reported and geospatial distance were associated with a decreased likelihood of using a bikeway (17). Moreover, King et al found that walking levels among older women were higher among those living in areas where parks or trails existed (10). Their study, however, did not specifically measure the impact of a walking trail. Nevertheless, they concluded that the ability to engage in walking trips from home and the perception of having favorable neighborhood surroundings for walking are associated with increased physical activity levels (10). Merom et al found that trail usage was increased among cyclists, particularly among individuals in close proximity to a trail (11). In our study, data suggest that convenient, safe, and proximal community walking trails provide an incentive for community residents to engage in regular physical activity. This offers further support to the importance of closely linking recreational trails with residential communities to provide safe and convenient access.

The type and pattern of physical activity on the trail also differed between new exercisers and habitually active individuals. It appears that newer exercisers begin with a more conservative physical activity (walking), whereas habitually active trail users more commonly select moderate- to high-intensity activities (e.g., running, in-line skating). Choosing more conservative physical activities like walking may also be related to a concern for personal safety and injury prevention. Both safety and terrain issues emerged as significant enablers for trail use among new exercisers. Consequently, new exercisers may be more concerned with injury prevention during physical activity and may use the trail because they feel it is safe and appropriate for exercise. Similarly, new exercisers were more likely to rate unsafe conditions as a barrier when asked, "What issues may prevent you from using the trail more frequently?" These data suggest that new exercisers are more sensitive to safety concerns than habitually active individuals.

How individuals perceive their environment may be more important in persuading a physically active lifestyle (18,19). Carnegie et al (20) identified a link between perceptions of the environment and stage of change for physical activity (21). In their study, contemplators (21) (inactive but intend to become more physically active) had more negative perceptions of the environment for physical activity. Similarly, it is reasonable to believe that the new exercisers in the present study were still embracing more negative perceptions of the environment than those who are habitually active. Developing strategies to address safety concerns along with other negative perceptions may be necessary if individuals are to progress to being habitually active. As such, trail advocates should prioritize and address safety concerns among new exercisers to promote the appeal of a trail for the long-term pursuit of enhancing physical activity within a community.

Although this preliminary investigation found that new exercisers appear to be more dependent on a recreational trail for achieving a pattern of regular physical activity compared with habitually active exercisers, this study has the following limitations:

- This investigation used a cross-sectional design that prohibited us from obtaining a baseline assessment of physical activity levels prior to the development of the trail.
- We relied on trail interviews, which may be subject to a potential response bias. Although we were unable to determine a true response rate, nearly all individuals (98%) approached on the trail were willing to participate.
- We used self-reported physical activity data, so there is no direct evidence that trail activities reported were actually performed. Nevertheless, every effort was made to conduct the interviews in a standardized format.
- The construct used to classify new vs habitual exercisers was not validated. We relied on individual recall. Consequently, it is possible that some trail users were misclassified. However, nearly all of the respondents (94%) were meeting physical activity recommendations (engaged in 150 minutes of leisure-time physical activity per week). Furthermore, to prevent a response bias, we asked participants about the type, frequency, and duration of their physical activity *before* asking them whether they were exercising regularly (more than three times per week for 20 minutes).
- Finally, we used an interceptor-based survey approach to probe respondents' views of the trail and identify their perceptions of the environment. Thus, while the information presented helps to identify perceptions of the environment for the trail user, it does not necessarily reflect the impact of the trail on the overall community. However, community-wide phone-survey data (unpublished data), which were obtained during the same time, indicate that 20% of regular exercisers use the trail as their primary exercise venue and only neighborhood streets provided a more common exercise location among community residents. Perhaps a lack of connectivity to the trail prevented many community members from using the trail as a primary site for regular physical activity. Given that there are very few walkable neighborhoods (e.g, no sidewalks, bike lanes, traffic-calming strategies) within the community, trail use would likely further increase if pedestrian connectivity from the trail to residential areas improved.

Regardless, these data provide a preliminary assessment of the importance of physical environmental changes, such as the development of a walking and biking trail, for promoting physically active lifestyles. Although a community trail can provide opportunities for all residents to engage in regular physical activity, both proximal and safe access from residential areas and safety on the trail may be important issues to encourage trail use among new exercisers.

## Figures and Tables

**Table 1:** Socio-demographic Characteristics of Trail Users (n = 414), Morgantown, WVa, 2001

**Characteristic**	**No. (%)**

**Sex**	

Female	228 (55.1)
Male	186 (44.9)

**Age (years)**	

18-25	96 (23.2)
26-35	92 (22.2)
36-45	89 (21.5)
46-65	110 (26.6)
65+	27 (6.5)

**Race/ethnicity**	

White	391 (94.4)
Black	7 (1.7)
Other	13 (3.1)
Declined	3 (0.7)

**Annual household income, $**	

<$10,000	111 (26.8)
$10,000 to 30,000	105 (25.4)
$31,000 to 60,000	114 (27.5)
>$60,000	54 (13.0)
Declined	30 (7.2)

**Education**	

High school/GED	145 (35.0)
Technical school	16 (3.9)
College graduate	160 (38.6)
Graduate school	61 (14.7)
Professional degree	30 (7.2)
Declined	2 (0.5)

**Employment Status**	

Homemaker	28 (6.8)
Self-employed	30 (7.2)
Student	100 (24.2)
Employed for wages	213 (51.4)
Retired	33 (8.0)
Unemployed	7 (1.7)
Declined/Other	3 (0.7)

**Table 2:** Perceived Enabling Factors and Personal Barriers to Trail Use for New Exercisers and Habitually Active Exercisers (n = 414), Morgantown, WVa, 2001

	**Habitually active (n = 321) **		**New exerciser (n = 93)**		**Difference between new and habitually active exercisers**

**Characteristic**	**Mean (±SD)^a^**	**Rank^b^**	**Mean (±SD)**	**Rank**	***P^c^***

**Enablers**					

Safety	3.9 (1.3)	4	4.2 (1.0)	3	.03
Scenery/environment	4.0 (1.0)	3	4.1 (1.0)	4	.16
Terrain (flat, paved)	4.3 (0.9)	1	4.6 (0.7)	2	.04
Convenience	4.3 (0.9)	2	4.7 (0.5)	1	<.001
Atmosphere	3.8 (1.1)	5	4.1 (1.2)	5	.19

**Barriers**					

Unsafe	2.7 (1.7)	3	3.1 (1.6)	2	.04
Parking	2.1 (1.3)	6	2.1 (1.4)	6	.78
Accessibility	2.2 (1.3)	5	2.4 (1.6)	5	.11
Facilities	3.1 (1.4)	1	3.4 (1.4)	1	.08
Maintenance	3.0 (1.5)	2	2.8 (1.6)	3	.28
Congestion	2.6 (1.4)	4	2.5 (1.4)	4	.53

a Mean values represent a five-point Likert scale ranging from 1 = not important at all to 5 = most important.

b Rank is based on the aggregate level of importance placed on each variable.

c Based on independent *t*-test.

## Appendix

**Recreational Trail Evaluation Survey, Morgantown, WVa, 2001**

Interviewer name:

Interview date:

Interview time:

Trailhead location:

Statement: Hello, We are conducting an interview about the recreational trail on behalf of the Division of Exercise Physiology and the Prevention Research Center at West Virginia University. We would like to get your opinions about the usage of the trail. The interview will take approximately five minutes to complete. Your responses are confidential and no identifying information will be obtained. Participation is voluntary and you may refuse to answer any questions.

1. Have you already been interviewed? Yes (Stop — not eligible) or No (Continue)
2. Would you like to participate? Yes (Continue to question 3) or no (Stop — not eligible)
3. Are you 18 or older? Yes (Continue to question 4) or no (Stop — not eligible)
4. How long have you been using the trail? (Weeks, months, or year)
5. What type of activity do you usually do on the trail? (Walk, run, bike, or inline skate)
6. How far do you usually perform [stated activity]? (Miles)
7. Where do you usually enter and exit the trail? (Caperton Trail: start, turn around, or finish) (Decker's Creek Trail: start, turn around, or finish)
8. How many minutes does this usually take you?
9. How many times (days) per week do you use the trail for [stated activity]?
10. Is there a second activity that you do on the trail? (If no, skip to 15)
11. How far do you usually perform [stated activity]? (Miles)
12. Where do you usually enter and exit the trail? (Caperton Trail: start, turn around, or finish) (Decker's Creek Trail: start, turn around, or finish)
13. How many minutes does this usually take you?
14. How many times (days) per week do you use the trail for [stated activity]?
15. Did you exercise regularly (three or more times per week for 20 minutes per session) before using this trail? Yes or no
16. a. Since using the trail, has the amount of exercise that you do: Increased (Skip to question 16b); decreased (why?); stayed the same; or don't knowb. Since using the trail, approximately how much has your exercise increased? (0–25%; 26–50%; 51–75%; 76–100%; over 100%)
17. On most days, where do usually come from to get to the trail? (Work, home, school, or other [identify other])
18. On most days how do you get to the trail? (Walk, drive, bicycle, bus, or other [identify other])
19. How far do you travel to use the trail? (Miles)
20. How long does it take you to get to the trail by walking? (Minutes)
21. While on the trail do you usually exercise: with others or alone (If alone, skip to question 23)
22. Who do you usually exercise with? (Friends, other family members/relatives, spouse/partner, walk/run club, children, pets, or other [identify other])
23. What time of the day do you usually use the trail? *[Read categories aloud]* Early morning (5–8:00 AM), Morning (8–11:00 AM), Midday (11:00 AM–2:00 PM), Afternoon (2-6:00 PM), Evening (after 6:00 PM), Varies, Refuse to answer
24. Please rate the following reasons on why you use this trail instead of other facilities on a scale of 1 to 5:
  **Table:**
  	**Least important = 1 to ** **most important = 5**				
  **Safety (free from personal injury) **	1	2	3	4	5
  **Scenery (beauty of environment)**	1	2	3	4	5
  **Access (no cost associated with use)**	1	2	3	4	5
  **Terrain (paved, flat)**	1	2	3	4	5
  **Convenience (location)**	1	2	3	4	5
  **Friendly atmosphere (social environment)**	1	2	3	4	5
  **Other (please identify)**	1	2	3	4	5
25. What is the primary reason why you use the trail instead of other facilities?
26. Please rate the following concerns you have about the trail on a scale of 1 to 5:
  **Table:**
  	**Least important = 1 to ** **most important = 5**				
  **Unsafe**	1	2	3	4	5
  **Parking (cost, lack of)**	1	2	3	4	5
  **Accessibility of the trail**	1	2	3	4	5
  **Facilities (restrooms, water fountains)**	1	2	3	4	5
  **Maintenance**	1	2	3	4	5
  **Space/congestion on the trail**	1	2	3	4	5
  **Fear of injury**	1	2	3	4	5
  **Lack of police patrol**	1	2	3	4	5
  **Visibility of distance/mile markers**	1	2	3	4	5
  **Other (please identify)**	1	2	3	4	5
27. What concerns you the most about the trail?
28. Apart from your trail activities, in the past month, have you participated in any of the following?
  **Table:**
  	**Yes**	**No**	**Number of ** **days per week**	**Minutes per ** **session**
  **Aerobic dance**				
  **Bicycling**				
  **Strength training**				
  **Golf**				
  **Jogging/running**				
  **Walking**				
  **Gardening**				
  **Swimming/water exercises**				
  **Organized team sports**				
  **Housework**				
  **Other**				
29. Age: 18–25, 26–35, 36–45, 46–65, 65 and above, declined to answer
30. Sex: Male, female, declined to answer
31. Race/ethnic origin: White, African American, Asian American, Hispanic, other (identify other), declined to answer
32. Employment status: Homemaker, self-employed, student, employed for wages, retired, unemployed, other (identify other), declined to answer
33. Educational attainment: Eighth grade or less, high school or GED, technical school, college graduate, graduate school, professional degree, declined to answer
34. Income level: Under $10,000; $10-30,000; $31-60,000; more than $60,000; declined to answer
